# Functional capacity testing in patients with pulmonary hypertension (PH) using the one-minute sit-to-stand test (1-min STST)

**DOI:** 10.1371/journal.pone.0282697

**Published:** 2023-03-09

**Authors:** Christina Kronberger, Roya Anahita Mousavi, Begüm Öztürk, Robin Willixhofer, Theresa-Marie Dachs, René Rettl, Luciana Camuz-Ligios, Nima Rassoulpour, Christoph Krall, Brigitte Litschauer, Roza Badr Eslam

**Affiliations:** 1 Department of Cardiology, Medical University of Vienna, Vienna, Austria; 2 Department of Cardiology, Clinic Favoriten, Vienna, Austria; 3 Center for Medical Data Science, Medical University of Vienna, Vienna, Austria; 4 Department of Clinical Pharmacology, Medical University of Vienna, Vienna, Austria; VA Boston Healthcare System, UNITED STATES

## Abstract

**Background:**

The one-minute sit-to-stand-test (1-min STST) is a quick, space saving test to evaluate functional capacity. Exercise testing plays an important role in the long-term follow-up of pulmonary hypertension (PH) patients and is currently evaluated using the six-minute-walk-test (6MWT). The aim of the study was to assess the convergent validity of the 1-min STST in patients with PH and its association with markers of PH severity.

**Methods:**

We evaluated 106 PH patients with the 1-min-STST and 6MWT and measured cardiorespiratory parameters (heart rate, blood pressure, oxygen saturation) before and after test conduction. N-terminal pro brain-type natriuretic peptide (NT-proBNP), WHO functional class (WHO-FC) and mean pulmonary artery pressure (mPAP) were defined as markers of PH severity.

**Results:**

Strong correlation was found between performances of 1-min STST and 6MWT (r = .711, *p* < .001), indicating convergent validity. Both tests were inversely associated with NT-proBNP (STST: r = -.405, *p* < .001; 6MWT: r = -.358, *p* < .001), WHO-FC (STST: r = -.591, *p* < .001; 6MWT: r = -.643, *p* < .001) and mPAP (STST: r = -.280, *p* < .001; 6MWT: r = -.250, *p* < .001). Significant changes in cardiorespiratory parameters were observed in both tests (all *p* < 0.001). Further the post-exercise cardiorespiratory parameters correlated strongly between the 1-min STST and 6MWT (all r ≥ .651, all *p* < .001).

**Conclusion:**

The 1-min STST demonstrated good convergent validity with the 6MWT and was associated with markers of PH severity. Furthermore, both exercise tests caused similar cardiorespiratory responses.

## Introduction

Pulmonary hypertension (PH) is a progressive disease defined by a mean pulmonary artery pressure (mPAP) ≥ 20 mmHg measured by right heart catheterization (RHC) [1], that, if untreated subsequently results in right heart failure and death.

With disease progression, patients develop worsening of dyspnea, fatigue, syncope and chest pain, which negatively impact functional capacity and quality of life.

The 6-minute walk test (6MWT) is a well-established method to assess functional capacity in patients with PH and is recommended by current European Society of Cardiology (ESC)/European Respiratory Society (ERS) guidelines for the diagnosis and treatment of PH [1]. However, it requires space and time and can therefore not always be performed in routine clinical practice.

Recent studies suggest the use of the one-minute sit-to-stand test (1-min STST) as an alternative to the 6MWT in several cardiopulmonary diseases [2–6]. In this test the participant is encouraged to stand up and sit down from a chair as quickly and as many times as possible within one minute. The 1-min STST is easier applicable, because it does not require as much space as the 6MWT. Therefore, we hypothesize that the 1-min STST can be used as a surrogate for the 6MWT in patients with PH as previously suggested by Nakazato et al. [7].

A good correlation of 1-min STST and 6MWT has already been shown in patients with chronic obstructive pulmonary disease (COPD) [8] and cystic fibrosis [3], however, little is known on the agreement of 1-min STST performance and 6MWT performance in patients with PH.

N-terminal prohormone of brain natriuretic peptide (NT-proBNP), WHO functional class (WHO-FC) and mean pulmonary artery pressure (mPAP) have been described as markers of PH severity [1, 9, 10]. The 6MWT was associated with PH severity markers in several studies [11–14], however, little is known about this association regarding the 1-min STST.

The aim of our study was to assess the convergent validity of the 1-min STST with the 6MWT in patients with PH and its association with markers of PH severity. Additionally, we wanted to analyze whether cardiorespiratory parameters respond similar to both exercise tests.

## Methods

### Study design

In this prospective, cross-sectional study data from PH patients at our specialized center were collected from March 2020 to July 2022. PH was defined as mPAP ≥ 20 mmHg invasively measured by right heart catheterization [15, 16] which is in accordance with the current ESC/ERS PH guidelines [1]. Patients performed the 1-min STST and 6MWT using a randomized test order by simple randomization method as described by Suresh et al. [17]. 46% of patients started with the 1-min STST and 54% started with the 6MWT followed by a resting period of 15 minutes as previously described [18]. All patients who were able to perform the two proposed exercise tests and gave their written informed consent were included in our study. The inability to perform one of both exercise tests (e.g.: due to physical impairment or mental illness) was our prespecified exclusion criterion. Further, patients with mPAP < 20 mmHg measured by right heart catheterization (n = 19) were excluded. Since we are a referral center for PH, patients were included into the study either at the time of their first visit or immediately before RHC during in-hospital stay. Timeframe of exercise testing to right heart catheterization was by median 0 months [IQR -3 to 0]. All patients underwent QoL assessment via the PH-specific Cambridge Pulmonary Hypertension Outcome Review (CAMPHOR) questionnaire [19] as well as WHO-FC assessment. Additionally, NT-proBNP levels were measured. Markers of PH severity were defined as NT-proBNP, WHO-FC and invasively measured mPAP. This study was approved by the Ethics Review Board of the Medical University of Vienna (EK#1123/2020) and complies with the principles stated in the Declaration of Helsinki.

### Test procedures

The 1-min STST was performed as described by previous studies [6, 20, 21]. A standardized chair with a seat height of 45 cm was used for the 1-min STST. The 6MWT was conducted according to the American Thoracic Society Statement guidelines [22]. Perceived dyspnea was recorded at the end of the 1-min STST and the 6MWT using the Borg Dyspnea Scale (BDS) [23]. Furthermore, cardiorespiratory parameters (blood pressure, heart rate and peripheral oxygen saturation) were measured before both tests at rest, immediately following test cessation as well as three minutes after test performance.

### Statistical analysis

Continuous variables were described by either arithmetic mean and standard deviation, or median and first and third quartiles depending on their distribution. Categorical variables were displayed with numbers and percentages. Convergent validity was examined through the investigation of the correlation between the number of 1-min STST repetitions and the six-minute walk distance (6MWD) using the Spearman’s rank correlation coefficient (r_s_). A correlation coefficient ≥ 0.5 was considered as supportive for convergent validity. The amount of agreement between the two tests was quantified using a Bland-Altman plot with standardized values (1-min STST results plotted against 6MWD using z-scores). Further, we analysed correlation of 1-min STST results and 6MWT results with PH severity markers. Differences between the correlation coefficients of the tests with PH severity markers were analysed according to Hittner [24] using Fisher´s Z-Transformation.

For analysis of cardiorespiratory response (oxygen saturation, heart rate, blood pressure) a repeated measures analysis of variance (ANOVA) was performed for the two tests and behaviour of each parameter under 1-min STST and 6MWT was compared in a multivariate analysis.

The correlation of patient’s perceived dyspnoea ratings after 1-min STST and 6MWT were assessed with the Spearman’s rank correlation coefficient. A two-sided *p*-value of less than .05 was defined as statistically significant.

Data were computed using IBM® Statistical Package for Social Sciences (SPSS)® version 26.0, New York, USA and R Statistical software version 4.2.2.

## Results

All clinical characteristics and test outcomes are displayed in **Table 1**.

**Table 1:** Clinical characteristics.

Variable		All patients (n = 106)		
**Baseline characteristics**				
Age (y)		66 ± 15		
Female sex		60 (57%)		
BMI (kg/m^2^)		28 ± 6.8		
Smoking (ex and current)		59 (56%)		
COPD		22 (21%)		
Diabetes mellitus		35 (33%)		
Coronary heart disease		28 (26%)		
Arterial hypertension		69 (65%)		
Hyperlipoproteinemia		66 (62%)		
Atrial fibrillation		47 (44%)		
**Test outcomes**					
1-min STST (reps.)		17 ± 7		
1-min STST % of predicted (reps.) ^1^		50 ± 18		
6MWD (meters)		351 ± 137		
6MWD % of predicted (m) ^2^		75 ± 40		
BDS end-STST		5.0 ± 2.3		
BDS end-6MWT		5.0 ± 2.7		
Quality of life score^✛^		27 ± 19		
**Markers of PH severity**					
NT-proBNP level (pg/mL)		1052 [232–2407]		
WHO FC I		11 (10%)		
WHO-FC II		37 (35%)		
WHO-FC III		52 (49%)		
WHO FC IV		6 (5.6%)		
**Right heart catheterization**					
mPAP (mmHg)		41 ± 14		
PCWP (mmHg)		15 ± 8.2		
LVEDP (mmHg) *		17 ± 6.6		
PVR (Wood units)		5.5 ± 3.9		
Saturation in the aorta (%)		91 ± 6.5		
Saturation in the pulmonary artery (%)		62 ± 9.6		

^1^ Based on the predicted values reported by Strassmann et al. [21]

^2^ Based on the reference values created by Trooster et al. [25]

^✛^ Quality of life scores were missing for 8 patients.

* LVEDP was not measured in 46 patients.

Unless otherwise indicated values are given as mean ± standard deviation, median (interquartile range) or absolute frequency and percentage frequency. Percentages may not total 100 because of rounding.

*Abbreviations*. y = years; BMI = body mass index; COPD = chronic obstructive pulmonary disease; 1-min STST = one-minute sit-to-stand test; reps. = repetitions; 6MWD = six-minute walk distance; BDS = Borg Dyspnea Score; STST = sit-to-stand test; 6MWT = six-minute walk test. NT-proBNP = N-terminal prohormone of brain natriuretic peptide; WHO-FC = World Health Organization (WHO) functional class; mPAP = mean pulmonary artery pressure; PCWP = pulmonary capillary wedge pressure; LVEDP = left ventricular end-diastolic pressure; PVR = pulmonary vascular resistance.

In total, 106 patients were included in our study. Mean age was 66 ± 15 years and 57% were females.

Mean 1-min STST repetition number was 17 ± 7 and mean 6MWT distance was 351 ± 137 meters. The mean BDS score reported after the 1-min STST was 5.0 ± 2.3 and 5.0 ± 2.7 after the 6MWT (*p* = .794).

The overall study population showed mild to moderate QoL impairment assessed with the CAMPHOR questionnaire (mean score 27 ± 19 points).

As for markers of PH severity, median NT-proBNP level was 1052 pg/mL [IQR: 232–2407] the majority of patients had WHO-FC III symptoms (n = 52, 49%) and mean mPAP was increased to 41 ± 14 mmHg. PH subtypes of the included patients are shown in **S2 Table**.

### 1-min STST vs. 6MWT

**Fig 1:**
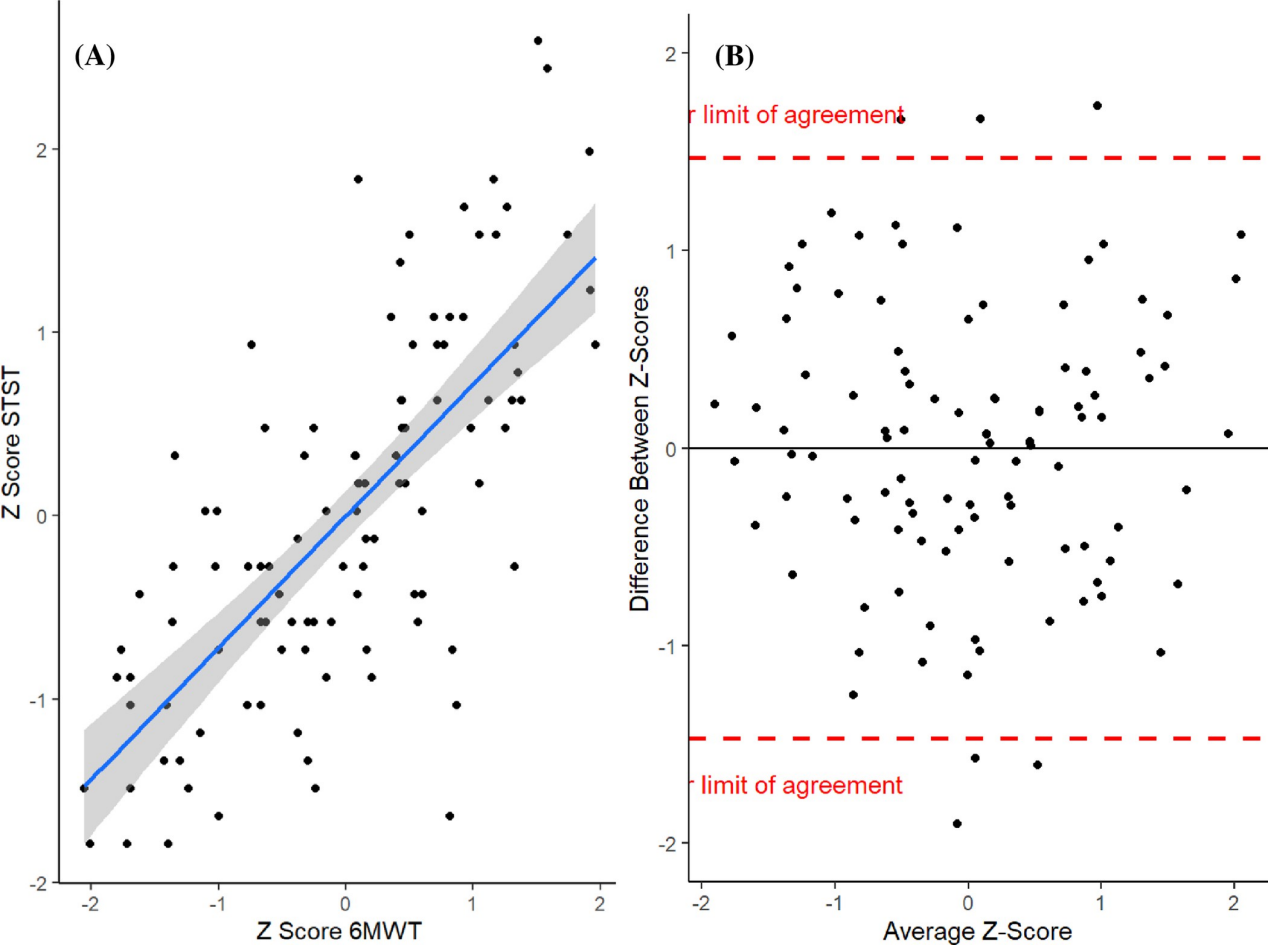
1-min STST versus 6MWT. **(A)** Correlation between z-scores of the 1-min STST and 6MWT and (**B)** corresponding Bland Altman plot. The solid line represents the mean difference of the methods (mean bias) and the dashed lines indicate the upper and lower limit of agreement. Filled circles represent individual measurements. *Abbreviations*. 1-min STST = one-minute sit-to-stand test; STST = sit-to-stand test; 6MWT = six-minute walk test.

1-min STST repetitions correlated strongly with 6MWD (r = .711, p < .001) as shown in **Fig 1**, panel A. The corresponding Bland Altman Plot was used to visualize the agreement between both tests (**Fig 1,** panel B).

The limits of agreement were calculated as mean difference ± 1.96 SD which are marked in the Bland Altmann Plot at +1.47 and -1.47. Seven (6.6%) patients fell outside the limits of agreement. A subsequent inspection of outliers was performed and is shown in **S1 Fig and S1 Table**.

### Markers of PH severity

Correlations of the two exercise tests and PH severity markers are displayed in **Table 2**.

**Table 2:** Correlations between exercise tests and PH severity markers.

	1-min STST		6MWT		1-min STST vs. 6MWT *
	r	*p*	r	*p*	*p*
**NT-proBNP**	-.405	**< .001**	-.358	**< .001**	**.549**
**WHO-FC**	-.591	**< .001**	-.643	**< .001**	**.561**
**mPAP**	-.280	**< .001**	-.250	**< .001**	**.895**

* Comparison of correlation coefficients of 1-min STST and 6MWT using Fisher´s Z-Transformation.

*Abbreviations*. 1-min STST = one-minute sit-to-stand test; 6MWT = six-minute walk test; NT-proBNP = N-terminal prohormone of brain natriuretic peptide; WHO-FC = World Health Organization functional class (modified after NYHA classification); mPAP = mean pulmonary artery pressure.

**Fig 2:**
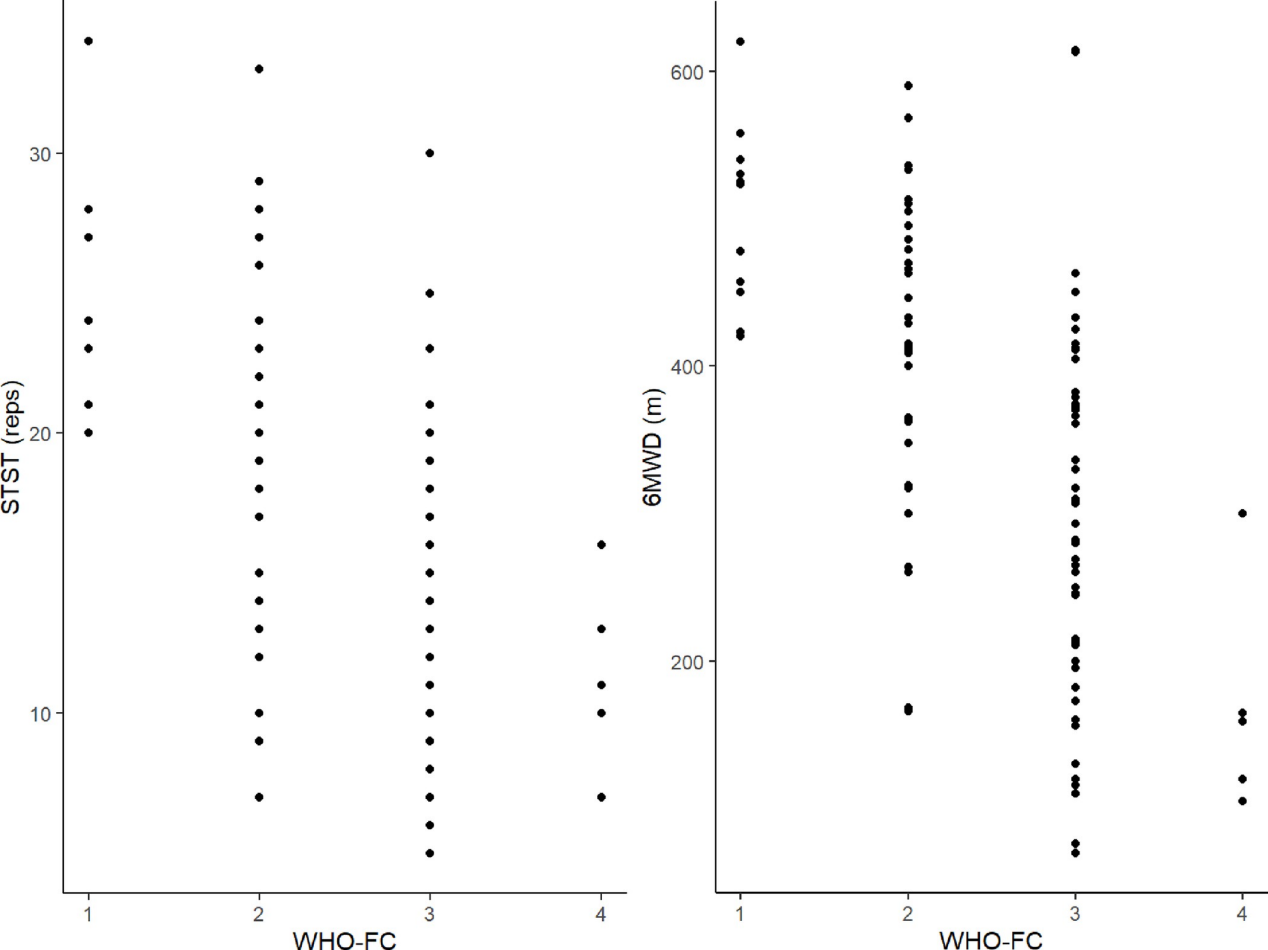
1-min STST and 6MWT performance in relation to WHO functional class. *Abbreviations*. 1-min STST = one-minute sit-to-stand test; reps = number of repetitions; 6MWD = six-minute walk distance, WHO-FC = World Health Organization functional class; STST = sit-to-stand test.

An inverse correlation was observed between the 1-min STST and NT-proBNP (r = -.405, *p* < .001) as well as 6MWD and NT-proBNP (r = -.358, *p* < .001). Further WHO-FC correlated with 1-min STST repetitions (r = -.591, *p* < .001) and 6MWD (r = -.643, *p* < .001), as visualized in **Fig 2**. Additionally, mPAP showed a negative correlation with 1-min STST repetitions (r = -.280, *p* < .001) and 6MWD (r = -.250, *p* < .001). No statistically significant differences between the correlation coefficients of the tests with PH severity markers were found (all *p* > .05).

### Cardiorespiratory responses

**Fig 3:**
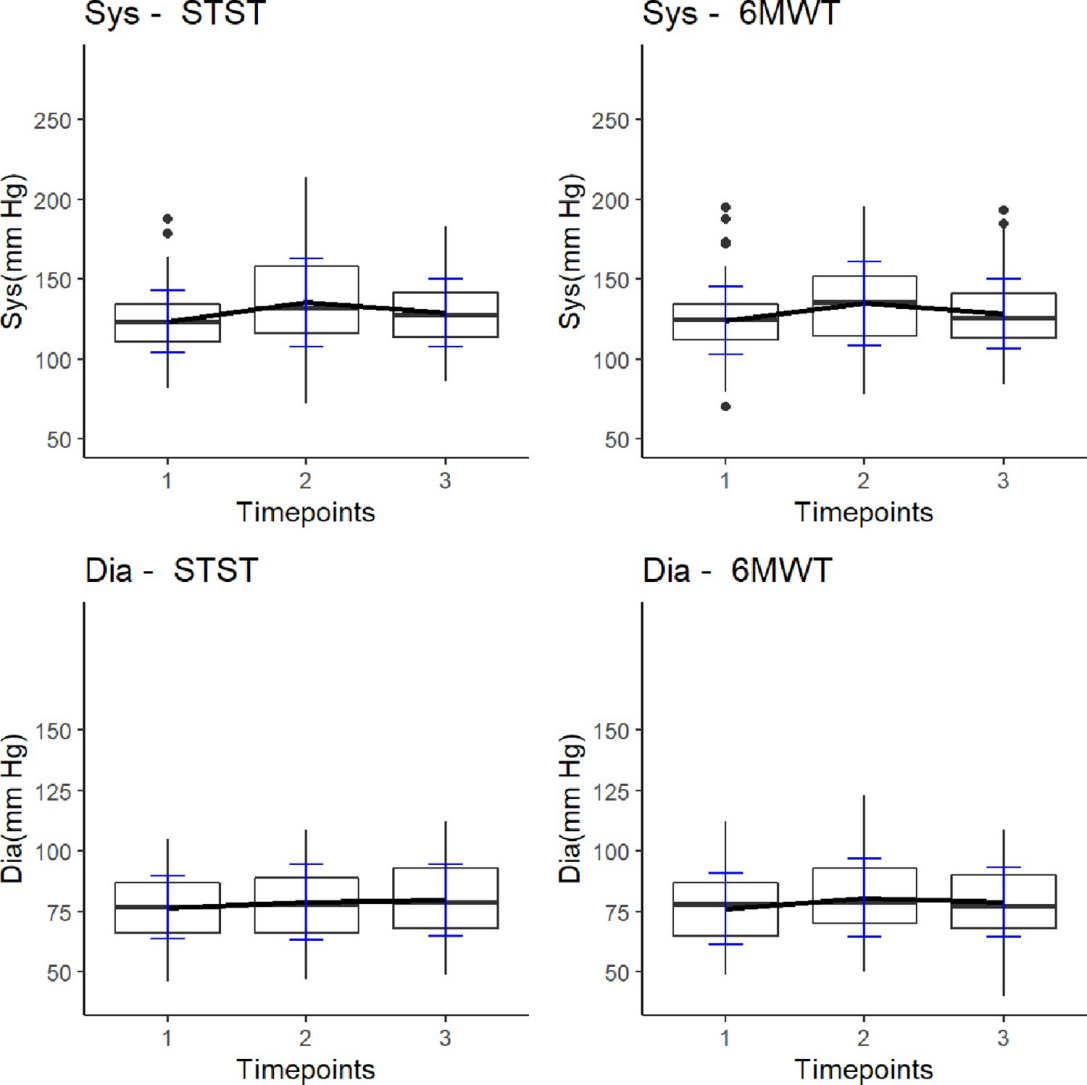
Line charts and box plots illustrating heart rate and SpO_2_ –STST versus 6MWT. Timepoints: 1 = before test, 2 = immediately after test performance, 3 = three minutes after test. Each box plot displays the following information: The heavy central line is the median value, the bottom and top lines of the box are the first and third quartiles of the data and individual dots are outlier data. *Abbreviations*. HR = heart rate; bpm = beats per minute; SpO_2_ = peripheral oxygen saturation; STST = sit-to stand test, 6MWT = six-minute walk test.

Cardiorespiratory responses of patients to the 1-min STST and 6MWT are shown in **Fig 3 and S2 Fig.**

Both tests lead to significant changes in cardiorespiratory parameters measured before, after and three minutes post-test conduction (all *p* < .001).

Systolic blood pressure increased on average 12 ± 20 mmHg during the 1-min STST and 11 ± 20 mmHg during the 6MWT (*p* = .933). Diastolic blood pressure showed a slight increase of 2.3 ± 10 mmHg during the 1-min STST, as opposed to a mean increase of 4.5 ± 12 mmHg during the 6MWT (*p* = .775). Both tests lead to heart rate changes with a mean increase of 21 ± 16 beats/min during the 1-min STST and 22 ± 18 beats/min during the 6MWT (*p* = .042). Oxygen saturation decreased by 4.6 ± 5.9% during the 1-min STST and 4.9 ± 6.5% during the 6MWT (*p* = .817). Except for heart rate, no statistically significant differences in cardiorespiratory responses between the 1-min STST and 6MWT were identified by multivariate repeated measures ANOVA. In correlation analysis the post-exercise cardiorespiratory parameters correlated strongly between the 1-min STST and 6MWT (all r ≥ .651, p < .001). Furthermore, BDS ratings recorded at the end of each exercise test showed significant correlation between both tests (r = .70, *p* < .001).

## Discussion

In our study, we were able to demonstrate agreement between the 1-min STST and the regularly used 6MWT in PH patients. To the best of our knowledge this is the first study exploring the convergent validity between 1-min STST performance and 6MWD in an overall PH cohort.

The main findings of our study are as follows: 1) the 1-min STST performance highly correlates with 6MWD in patients with PH and therefore convergent validity can be assumed, 2) the 1-min STST performance is associated with markers of PH severity (NT-proBNP, WHO-FC, mPAP) and 3) blood pressure and oxygen saturation responses do not differ significantly between the 1-min STST and 6MWT.

In our study a strong positive correlation between the number of 1-min STST repetitions with 6MWD was shown (*p* < .001). This is in line with findings of prior studies that showed moderate to strong correlations of the 1-min STST with the 6MWT in idiopathic pulmonary fibrosis, COPD, and cystic fibrosis populations [5, 26, 27].

Additionally, Bland Altman plot showed good agreement between both test methods with low variance in our study. This indicates that both tests can be used to assess functional capacity of PH patients.

### Markers of PH severity

NT-proBNP and WHO-FC are an integral component of risk assessment in PH and are included in the multiparametric risk stratification model outlined in the 2022 ESC/ERS guidelines [1]. Our study showed that both, the 1-min STST and the 6MWT, are associated with NT-proBNP and WHO-FC. An association between 6MWD and NT-proBNP as well as WHO-FC has been previously shown in patients with congestive heart failure [12, 13]. However, to the best of our knowledge, this is the first study that analyzes the association of 1-min STST performance with NT-proBNP and WHO-FC in patients with PH.

Our study found no statistically significant differences between the correlation coefficients of both exercise tests. This suggests that both the 6MWT and the 1-min STST may be equally used as indices for clinical manifestation of PH.

NT-proBNP and WHO-FC have been described as prognostic markers in patients with PH [10, 28, 29] and are associated with increased mortality [10, 29, 30]. The in our study observed correlation of 1-min STST performance with NT-proBNP and WHO-FC led us to assume that patients with worse 1-min STST performance may have worse prognosis. However, further studies are required to evaluate the role of the 1-min STST in this regard since we did not include long-term outcome data in our study.

Further, we found a negative correlation of the 1-min STST and 6MWT with mPAP.

Recent studies described the importance of mPAP values in the therapeutic management of patients with PH [9, 31, 32] and lowering of mPAP resulted in favorable outcomes in idiopathic PH patients [32]. Guidelines for the treatment of PH [31] consider a normalization of mPAP as a goal of treatment in heritable and idiopathic PH subtypes.

Our data suggests that the 1-min STST may be a good tool to identify patients with a worse clinical presentation of PH as it correlates with NT-proBNP, WHO-FC and mPAP. In our opinion an adaption of medical therapy may be considered in patients with a decrease in exercise test performances during follow-up examinations.

### Cardiorespiratory responses during test performance

Our study found no statistically significant differences in cardiorespiratory responses (blood pressure, oxygen saturation) between the 1-min STST and 6MWT. This indicates that both exercise tests are similar demanding and lead to comparable physical stress levels.

Further we found a strong correlation of patients’ dyspnea scores at test-end, which also demonstrates the comparability of physical demand in both tests.

Additionally, we noted a significant difference in heart rate response between the two tests (*p* = .042) in multivariate repeated measures ANOVA, though, the mean heart rate values post-tests only differed by two beats per minute. However, we also demonstrated that the post-exercise heart rate correlated strongly between the 1-min STST and 6MWT.

The comparison of cardiorespiratory responses during the 1-min STST and the 6MWT in previous studies showed conflicting results. In some studies, the cardiovascular demand seemed to be higher during the 6MWT than the 1-min STST [26, 33] which is contrary to findings of our study. However, those studies had a smaller sample size and evaluated a different study population. Other publications have demonstrated a comparable heart rate response in both tests, though blood pressure parameters were not evaluated [2, 34].

We assume, that the discordance regarding cardiorespiratory responses between those studies may be due to the variation in evaluated patient populations as well as different underlying diseases and sample sizes. Our data suggests that both tests lead to similar cardiorespiratory responses, which strengthens our hypothesis that the 1-min STST can be used as an alternative to the 6MWT in PH patients.

### 1-min STST vs. 6MWT

Currently, the 6MWT is commonly used to assess functional capacity in PH patients and is recommended by guidelines [1, 22]. The convergent validity of 1-min STST repetitions with 6MWD that was shown in our study suggests that the 1-min STST can also be used to assess functional capacity in patients with PH.

The 1-min STST is considered as a potential alternative to the 6MWT as it has several advantages [4, 8]. First, in comparison to the 6MWT the 1-min STST is less time and space consuming and can therefore be performed in all hospitals and diagnostic centers. Second, oxygen apparatuses, if needed, do not have to be carried by the probands during test procedure which makes the 1-min STST easier applicable in patients with pulmonary diseases.

As in our study the 1-min STST performance was significantly decreased with increasing PH severity markers, we postulate that the 1-min STST can be used as alternative test to the 6MWT in clinical practice to identify vulnerable PH patients. The good correlation shown in our study is in line with findings of a previous study that investigated the association of physical activity level with clinical factors and functional capacity in 20 PH patients [7].

However, the 1-min STST may not be optimal for patients with musculoskeletal limitations including knee- or hip problems, as shown by the outliers observed in our study (**S1 Table**). Further studies are required to evaluate the convergent validity of the 1-min STST with the 6MWT in patients with musculoskeletal issues and to define reference values for the 1-min STST in patients with PH.

We suppose that the 1-min STST could be particularly useful when the 6MWT is not applicable such as in the primary health care sector, where limited space, restricted time and minimum personnel resources prevent the application of the 6MWT.

### Study limitations

There are several limitations to our study that need to be taken into consideration. First, our study is limited by the fact that it is a single-center study. However, the test procedure and clinical routine were the same for every patient, which increases the comparability of our data. Second, this is a cross-sectional study, and it does not contain longitudinal data, therefore our data cannot be used for predictive conclusions. However, the prospective study design and randomized test order increase the accuracy of our study results.

Third, we used a standardized chair, thus, no adaption to patient’s leg length was done. However, this was analogous to other studies [20, 21, 35, 36] and facilitates the comparability of 1-min STST results between studies.

## Conclusion

The 1-min STST correlates with the 6MWT and is associated with markers of PH severity. Therefore, convergent validity can be assumed. The 1-min STST induces similar cardiorespiratory responses as the 6MWT and can thus be considered as an alternative tool to evaluate functional capacity in patients with PH.

## Supporting information

S1 Fig Bland-Altman plot with outliers numbered. (DOCX)

S2 Fig Line charts and box plots illustrating blood pressure in patients before, after and three minutes after both tests. (DOCX)

S1 Table Outliers with brief description of their test performance. (DOCX)

S2 Table Subtypes of PH. (DOCX)

## Abbreviation list

1-min STST: One-minute sit-to-stand test
6MWD: Six-minute walk distance
6MWT: Six-minute walk test
BDS: Borg Dyspnea Scale
BMI: Body mass index
CAMPHOR: Cambridge Pulmonary Hypertension Outcome Review
COPD: Chronic obstructive pulmonary disease
ESC: European Society of Cardiology
ERS: European Respiratory Society
mPAP: Mean pulmonary arterial pressure
NT-proBNP: N-terminal prohormone of brain natriuretic peptide
QoL: Quality of life
RHC: Right heart catheterization
WHO-FC: WHO functional class
