# Correction: Functional capacity testing in patients with pulmonary hypertension (PH) using the one-minute sit-to-stand test (1-min STST)

**DOI:** 10.1371/journal.pone.0317857

**Published:** 2025-01-16

**Authors:** Christina Kronberger, Roya Anahita Mousavi, Begüm Öztürk, Robin Willixhofer, Theresa-Marie Dachs, René Rettl, Luciana Camuz-Ligios, Nima Rassoulpour, Christoph Krall, Brigitte Litschauer, Roza Badr Eslam

The Fig 3 is uploaded incorrectly. The authors have provided correct version of Fig 3 here.

**Fig 3:**
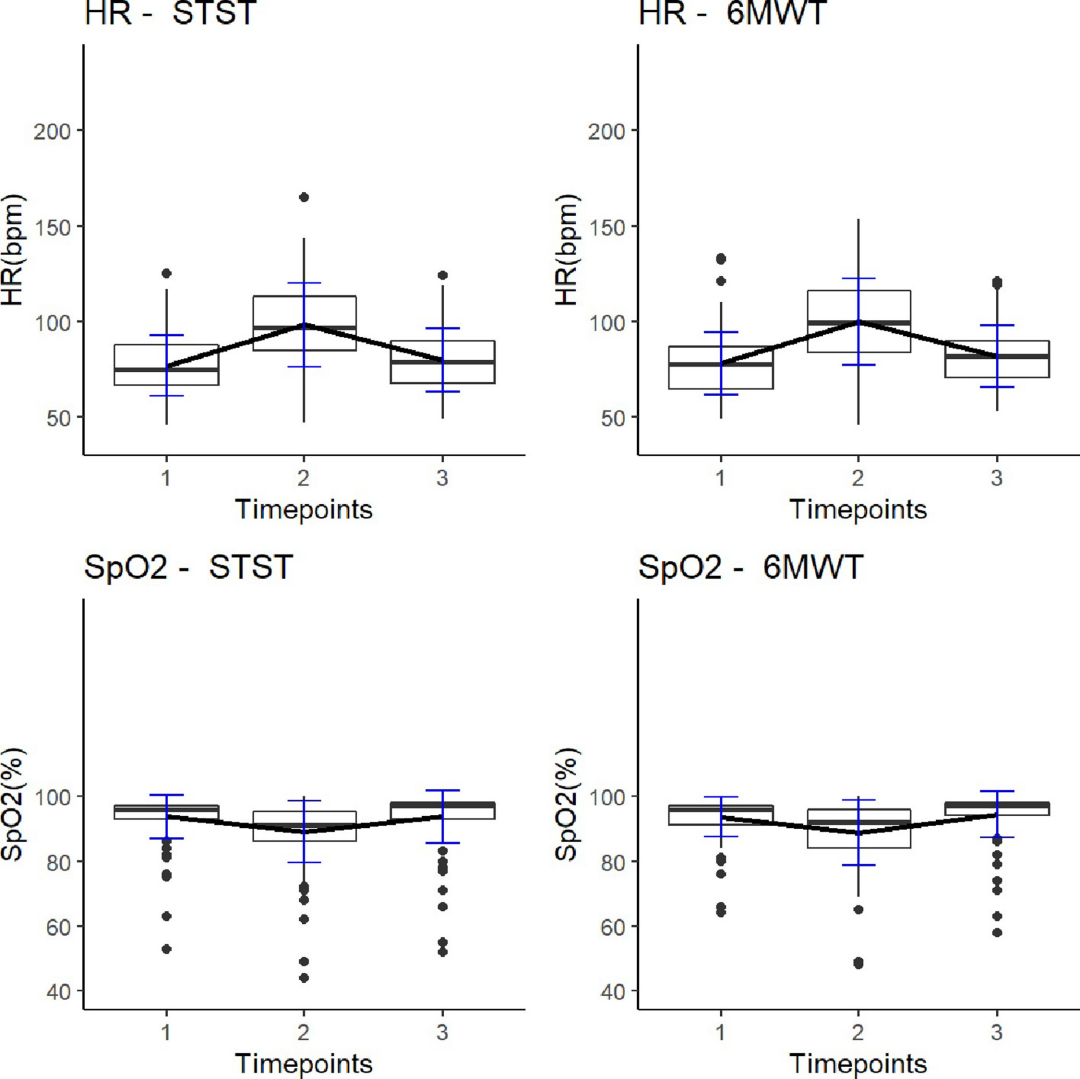
Line charts and box plots illustrating heart rate and SpO2 –STST versus 6MWT. Timepoints: 1 = before test, 2 = immediately after test performance, 3 = three minutes after test. Each box plot displays the following information: The heavy central line is the median value, the bottom and top lines of the box are the first and third quartiles of the data and individual dots are outlier data. Abbreviations. HR = heart rate; bpm = beats per minute; SpO2 = peripheral oxygen saturation; STST = sit-to stand test, 6MWT = six-minute walk test.Timepoints: 1 = before test, 2 = immediately after test performance, 3 = three minutes after test. Each box plot displays the following information: The heavy central line is the median value, the bottom and top lines of the box are the first and third quartiles of the data and individual dots are outlier data. Abbreviations. HR = heart rate; bpm = beats per minute; SpO2 = peripheral oxygen saturation; STST = sit-to stand test, 6MWT = six-minute walk test.
