# Supplementary figures and images for: Functional capacity testing in patients with pulmonary hypertension (PH) using the one-minute sit-to-stand test (1-min STST)
Source: PLoS One. 2023 Mar 9;18(3):e0282697. doi: 10.1371/journal.pone.0282697 (PMC9997887; doi:10.1371/journal.pone.0282697)

**S1 Fig.** *Bland-Altman plot with outliers numbered*


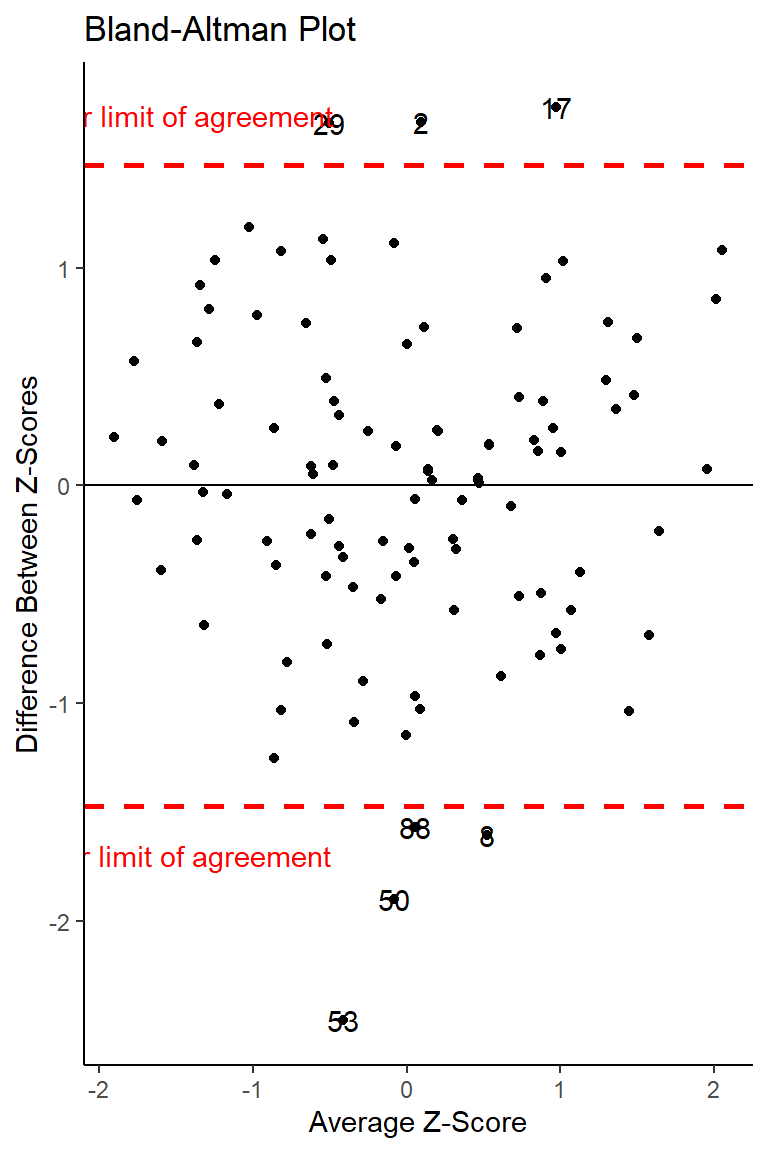

Supplement: S1 Fig — (DOCX) [file pone.0282697.s001.docx]
