# Supplementary material for: Functional capacity testing in patients with pulmonary hypertension (PH) using the one-minute sit-to-stand test (1-min STST)
Source: PLoS One. 2023 Mar 9;18(3):e0282697. doi: 10.1371/journal.pone.0282697 (PMC9997887; doi:10.1371/journal.pone.0282697)
Supplement: S2 Fig — (DOCX) [file pone.0282697.s002.docx]

**S2 Fig.** *Line charts and box plots illustrating blood pressure in patients before, after and three minutes after both tests*


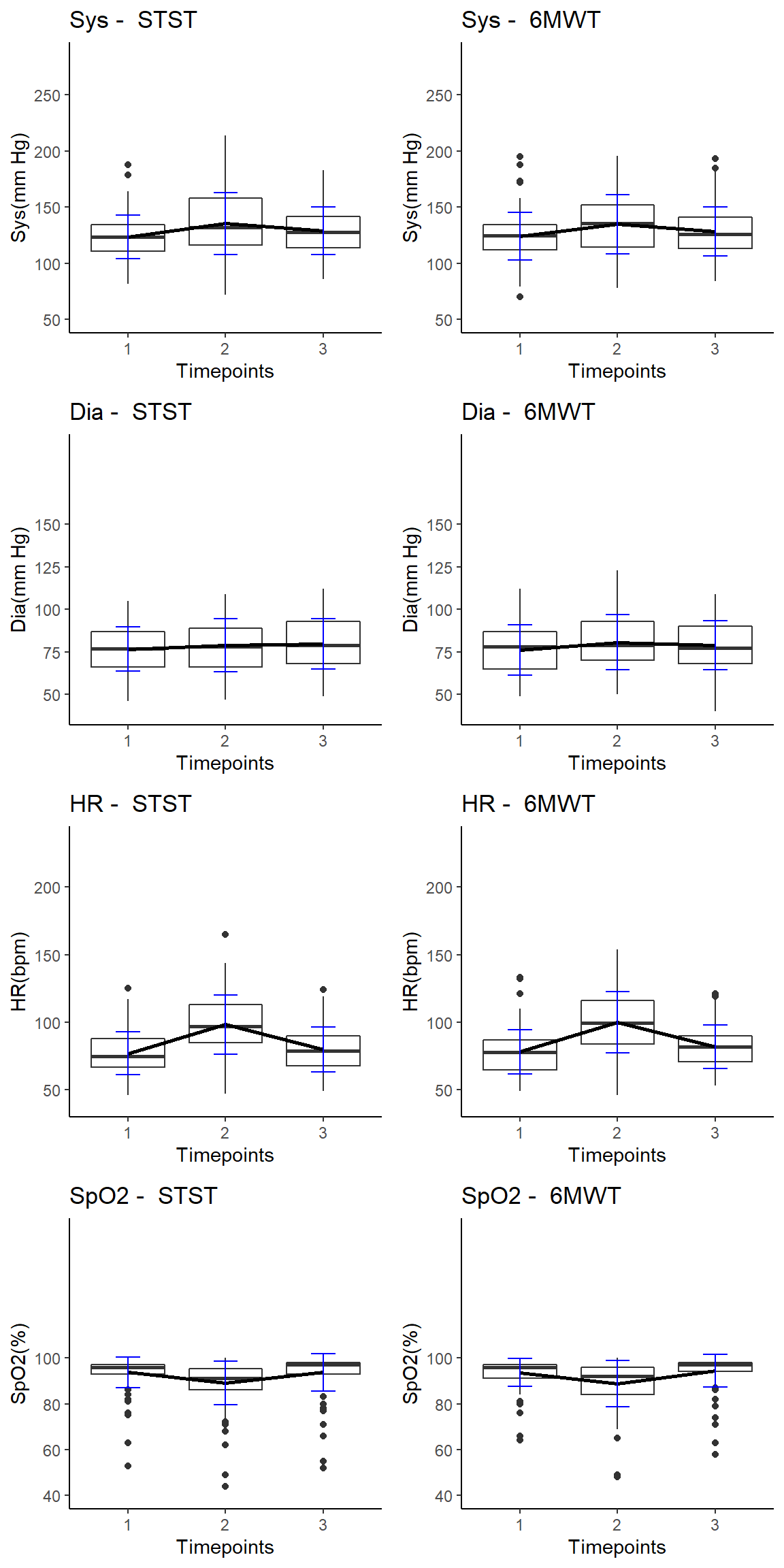


Timepoints: 1 = before test, 2 = immediately after test performance, 3 = three minutes after test

Each box plot displays the following information: The heavy central line is the median value, the bottom and top lines of the box are the first and third quartiles of the data and individual dots are outlier data.

*Abbreviations.* Sys = systolic blood pressure; Dia = diastolic blood pressure; STST = sit-to-stand-test; 6MWT = six-minute walk test.
