# Supplementary material for: Functional capacity testing in patients with pulmonary hypertension (PH) using the one-minute sit-to-stand test (1-min STST)
Source: PLoS One. 2023 Mar 9;18(3):e0282697. doi: 10.1371/journal.pone.0282697 (PMC9997887; doi:10.1371/journal.pone.0282697)
Supplement: S1 Table — (DOCX) [file pone.0282697.s003.docx]

**S1 Table**. *Outliers with brief description of their test performance*

| **No.** | **1-min STST, reps.** | **6MWD, m** | **Comment** |
| --- | --- | --- | --- |
| 2 | 23 | 250 | Interruption after three minutes, Oxygen device (2 liters of oxygen per minute) |
| 8 | 15 | 533 | Knee pain, mPAP 56 mmHg |
| 17 | 29 | 365 | Abdominal pain, had to stop several times |
| 29 | 19 | 168 | No information available |
| 50 | 10 | 470 | Hip dysplasia as a child and femoroacetabular impingement, takes the dog for a walk every day |
| 53 | 6 | 463 | Muscular weakness, had problems with standing-up |
| 88 | 12 | 466 | No information available |

*Abbreviations.* No. = number; 1-min STST = one-minute sit-to-stand test; reps. = number of repetitions; 6MWD = six-minute walk distance; m = meters; mPAP = mean pulmonary artery pressure.

*Note.* Number 8 and 50 struggled with knee respectively hip joint pain. This could explain why these patients showed relatively weak performance in the 1-min STST.
