# Supplementary material for: Functional capacity testing in patients with pulmonary hypertension (PH) using the one-minute sit-to-stand test (1-min STST)
Source: PLoS One. 2023 Mar 9;18(3):e0282697. doi: 10.1371/journal.pone.0282697 (PMC9997887; doi:10.1371/journal.pone.0282697)
Supplement: S2 Table — (DOCX) [file pone.0282697.s004.docx]

**S2 Table**. *Subtypes of PH*

| **PH subtypes – n (%)** |  |
| --- | --- |
| PAH | 13 (12%) |
| Left heart disease | 40 (38%) |
| Lung disease | 12 (11%) |
| CTEPH | 25 (24%) |
| Unclear and/or multifactorial cause | 16 (15%) |

*Abbreviations.* PH = pulmonary hypertension; PAH = pulmonary arterial hypertension; CTEPH = chronic thromboembolic pulmonary hypertension.
